# Supplementary material for: Heat adaptation of phage T7 under an extended genetic code
Source: Virus Evol. 2021 Dec 1;7(2):veab100. doi: 10.1093/ve/veab100 (PMC8923235; doi:10.1093/ve/veab100)
Supplement: veab100_Supp [file veab100_supp.zip › Sequencing_Analysis.zip!/Sequencing_Analysis/S3/evidence/JC_SIDE_2_49.html]

BRESEQ :: Evidence


| New junction evidence | | | | | | | | | | | |
| --- | --- | --- | --- | --- | --- | --- | --- | --- | --- | --- | --- |
|  | | seq id | position | reads (cov) | reads (cov) | score | skew | freq | annotation | gene | product |
| \* | ? | NC\_001604.1 | 8888 = | 2048 (1.190) | 3 (0.000) | 3/592 | 47.8 | 0.15% | intergenic (–/–) | *–/–* | –/– |
|  | ? | NC\_001604.1 | 8897 = | 2013 (1.190) |  |  |  |  | intergenic (–/–) | *–/–* | –/– |
| Rejected: Coverage evenness skew score above cutoff. | | | | | | | | | | | |
| Rejected: Frequency below/above cutoff threshold. | | | | | | | | | | | |

Only 100 of 2047 total aligned reads displayed.  

`GACCGTTGAGCAGTTCAAGGGATACTGCTTCGGTAACATCTTAAAGTACAGACTACGTGCTGGTAAGAAGTCAGAGTTAGCGTACTTAGAGAAAGACCTAGCGAAAGCAGACTTCTATAAAGAACTCTTTGAGAAACATAAGGATAAATGTTATGCATAACTTCAAGTCAA‑CCCCACCTGCCGACAGCCTATCTGATGACTTCACATCTTGCTCAGAGTGGTGCCGAAAGATGTGGGAAGAGACATTCGACGATGCGTACATCAAGCTGTATGAACTTTGGAAATCGAGAGGTCAATGACTATGTCAAACGTAAATACAGGTTCACTTAGTGTGGACAATAAGAAGTTTTGGGCTACCGTAGAGTCCTCGGAGCATTCCTTCGAGGTTCCAATCTACGCTGAGACCCTAGACGAAGCTCTGGAGTTAGCCGAATGGCAATACGTTCCGGCTGGCTTTGAGGTTACTCGTGTGCGTCCTTGTGTAGCACCGAAGTAATACGACTCACTATTAGGGAAGACTCCCTCTGAGAAACCAAACGAAACCTAAAGGAGATTAACATTATGGCTAAGAAGATTTTCACCTCTGCGCTGGGTACCGCTG  >  NC_001604.1/8597‑9197`  

                                                                                                                                                                                                                                                                                                             `|`                                                                                                                                                                                                                                                                                                              
      `agacagggtCAAGGGAGACTGCTTCGGTAACATCTTAAAGTACAGACTACGTGCTGGTAAGAAGTCAGAGTTAGCGTACTTAGAGAAAGACCTAGCGAAAGCAGACTTCTATAAAGAACTCTTTGAGAAACATAAGGATAAATGTTATGCATAACTTCAAGTCAA‑CCCCACCTGCCGACAGCCTATCTGATGACTTCACATCTTGCTCAGAGTGGTGCCGAAAGATGTGGGAAGAGACATTCGACGATGCGTACATCAAGCTGTATGAACTTTGGAAATCGAGAGGTCAATGACTATGTCa                                                                                                                                                                                                                                                                                                        <  1:201428/293‑1 (MQ=255)`  
          `gaGTTAAAGGGATACTGCTTCGGTAACATCTTAAAGTACAGACTACGTGCTGGTAAGAAGTCAGAGTTAGCGTACTTAGAGAAAGACCTAGCGAAAGCAGACTTCTATAAAGAACTCTTTGAGAAACATAAGGATAAATGTTATGCATAACTTCAAGTCAA‑CCCAACCTGCCGACAGCCTATCTGATGACTTCACATCTTGCTCAGAGTGGTGCCGAAAGATGTGGGAAGAGACATTCGACGATGCGTACATCAAGCTGTATGAACTTTGGAAATCGAGAGGTCAATGACTATGTCAAACg                                                                                                                                                                                                                                                                                                    <  1:33793/300‑1 (MQ=255)`  
          `cAGTTCAAGGGATACTGCTTCGGTAACATCTTAAAGTACAGACTACGTGCTGGTAAGAAGTCAGAGTTAGCGTACTTAGAGAAAGACCTAGCGAAAGCAGACTTCTATAAAGAACTCTTTGAGAAACATAAGGATAAATGTTATGCATAACTTCAAGTCAA‑CCACACCTGCCGACAGCCTATCTGATGACTTCACATCTTGCTCAGAGTGGTGCCGAAAGATGTGGGAAGAGACATTCGACGATGCGGATATCAAGCTGTATGAACTTTGGAAATCGAGAGGTCAATGACTATGTCAAACg                                                                                                                                                                                                                                                                                                    >  1:89253/1‑301 (MQ=255)`  
            `gTTCAAGGGATACTGCTTCGGTAACATCTTAAAGTACAGACTACGTGCTGGTAAGAAGTCAGAGTTATCGTACTTAGAGAAAGACCTAGCGAAAGCAGACTTCTATAAAGAACTCTTTGAGAAACATAAGGATAAATGTTATGCATAACTTCAAGTCAA‑CCCCACCTGCCGACAGCCTATCTGATGACTTCACATCTTGCTCAGAGTGGTGCCGAAAGATGTGGGAAGAGACATTCGACGATGCGTACATCAAGCTGTATGAACTTTGGAAATCGAGAGGTCAATGACTATGTCAAACGTa                                                                                                                                                                                                                                                                                                  >  1:227687/1‑301 (MQ=255)`  
            `gTTCAAGGGATACTGCTTCGGTAACATCTTAAAGTACAGACTACGTGCTGGTAAGAAGTCAGAGTTAGCGTACTTAGAGAAAGACCTAGCGAAAGCAGACTTCTATAAAGAACTCTTTGAGAAATATAAGGATAAATGTTATGCATAACTTCAAGTCAA‑CCCCACCTGCCGACAGCCTATCTGATGACTTCACATCTTGCTCAGAGTGGTGCCGAAAGATGTGGGAAGAGACATTCGACGATGCGTACATCAAGCTGTATGAACTTTGGAAATCGAGAGGTCAATGACTATGTCAAACGTa                                                                                                                                                                                                                                                                                                  >  1:282674/1‑301 (MQ=255)`  
            `gTTCAAGGGATACTGCTTCGGTAACATCTTAAAGTACAGACTACGTGCTGGTAAGAAGTCAGAGTTAGCGTACTTAGAGAAAGACCTAGCGAAAGCAGACTTCTATAAAGAACTCTTTGAGAAACATAAGGATAAATGTTATGCATAACTTCAAGTCAA‑CCCCACCTGCCGACAGCCTATCTGATGACTTCACATCTTGCTCAGAGTGGTGCCGAAAGATGTGGGAAGAGACATTCGACGATGCGTACATCAAGCTGTATGAACTTTGGAAATCGAGAGGTCAATGACTATGTCAAACGTa                                                                                                                                                                                                                                                                                                  >  1:190432/1‑301 (MQ=255)`  
                 `aGGGATACTGCTTCGGTAACATCTTAAAGTACAGACTACGTGCTGGTAAGAAGTCAGAGTTAGCGTACTTAGAGAAAGACCTAGCGAAAGCAGACTTCTATAAAGAACTCTTTGAGAAACATAAGGATAAATGTTATGCATAACTTCAAGTCAA‑CCCCACCTGCCGACAGCCTATCTGATGACTTCACATCTTGCTCAGAGTGGTGCCGAAAGATGTGGGAAGAGACATTCGACGATGCGTACATCAAGCTGTATGAACTTTGGAAATCGAGAGGTCAATGACTATGTCAAACGTAAATAc                                                                                                                                                                                                                                                                                             <  1:164132/301‑1 (MQ=255)`  
                 `aGGGATACTGCTTCGGTAACATCTTAAAGTACAGACTACGTGCTGGTAAGAAGTCAGAGTTAGCGTACTTAGAGAAAGACCTAGCGAAAGCAGACTTCTATAAAGAACTCTTTGAAAAACATAAGGATAAATGTTATGCATAACTTCAAGTCAA‑CCCCACCTGCCGACAGCCTATCTGATGACTTCACATCTTGCTCAGAGTGGTGCCGAAAGATGTGGGAAGAGACATTCGACGATGCGTACATCAAGCTGTATGAACTTTGGAAATCGAGAGGTCAATGACTATGTCAAACGTAAATAc                                                                                                                                                                                                                                                                                             <  1:123522/301‑1 (MQ=255)`  
                 `aGGGATACTGCTTCGGTAAAATCTTAAAGTACAGACTACGTGCTGGTAAGAAGTCAGAGTTAGCGTACTTAGAGAAAGACCTAGCGAAAGCAGACTTCTATAAAGAACTCTTTGAGAAACATGAGGATAAATGTTATGCATAACTTCAAGTCAA‑CCCCACCTGCCGACAGCCTATCTGATGACTTCACATCTTGCTCAGAGTGGTGCCGAAAGATGTGGGAAGAGACATTCGACGATGCGTACATCAAGCTGTATGAACTTTGGAAATCGAGAGGTCAATGACTATGTCAAACGTAAATAc                                                                                                                                                                                                                                                                                             <  1:270597/301‑1 (MQ=255)`  
                          `gCTTCGGTAACATCTTAAAGTACAGACTACGTGCTGGTAAGAAGTCAGAGTTAGCGTACTTAGAGAAAGACCTAGCGAAAGCAGACTTCTATACAGAACTCTTTGAGAAACATAAGGATAACTGTTATGCATAACTTCACGTCAA‑CCCCACCTGCCGACAGCCTATCTGATGACTTCACATCTTGCTCAGAGTGGTGCCGAAAGATGTGGGAAGAGACATTCAACGATGCGTACCTCAAGCTGTATGAACTTTGGAAATCGAGAGGTCAATGACTATGTCAAACGTACATACAGTTTCACt                                                                                                                                                                                                                                                                                    >  1:288767/1‑301 (MQ=255)`  
                          `gCTTCGGTAACATCTTAAAGTACAGACTACGTGCTGGTAAGAAGTCAGAGTTAGCGTACTTAGAGAAAGACCTAGCGAAAGCAGACTTCTATAAAGAACTCTTTGAGAAACATAAGGATAAATGTTATGCATAACTTCAAGTCAA‑CCCCACCTGCCGACAGCCTATCTGATGACTTCACATCTTGCTCAGAGTGGTGCCGAAAGATGTGGGAAGAGACATTCGACGATGCGTACATCAAGCTGTATGAACTTTGGAAATCGATAGGTCAATGACTATTTCAACCGTAAATACAGGTTCACt                                                                                                                                                                                                                                                                                    >  1:203420/1‑301 (MQ=255)`  
                            `ttCGGTAACATCTTAAAGTACAGACTACGTGCTGGTAAGAAGTCAGAGTTAGCGTACTTAGAGAAAGACCTAGCGAAAGCAGACTTCTATAAAGAACTCTTTGAGAAACATAAGGATAAATGTTATGCATAACTTCAAGTCAA‑CCCCACCTGCCGACAGCCTATCTGATGACTTCACATCTTGCTCAGAGTGGTGCCAAAAGATGTGGGAAGAGACATTCGACGATGCGTACATCAAGCTGTATGAACTTTGGAAATCGAGAGGTCAATGACTATGTCAAACGTAAATACAGGTTCACTTa                                                                                                                                                                                                                                                                                  >  1:335785/1‑301 (MQ=255)`  
                              `cGGTAACATCTTAAAGTACAGACTACGTGCTGGTAAGAAGTCAGAGTTAGCGTACTTAGAGAAAGACCTAGCGAAAGCAGACTTCTATAAAGAACTCTTTGAGAAACATAAGGATAAATGTTATGCATAACTTCAAGTCAA‑CCCCACCTGCCGACAACCTATCTGATGACTTCACATCTTGCTCAGAGTGGTGCCGAAAGATGTGGGAAGAGACATTCGACGATGCGTACATCAAGCTGTATGAACTTTGGAAATCGAGAGGTCAATGACTATGTCAAACGTAAATACAGGTTCAATTAgt                                                                                                                                                                                                                                                                                >  1:23719/1‑301 (MQ=255)`  
                                    `aagagacaggTACAGACTACGTGCTGGTAAGAAGTCAGAGTTAGCGTACTTAGAGAAAGACCTAGCGAAAGCAGACTTCTATAAAGAACTCTTTGAGAAACATAAGGATAAATGTTATGCATAACTTCAAGTCAA‑CCCCACCTGACGACAGCCTATCTGATGACTTCACATCTTGCTCAGAGTGGTGCCGAAAGATGTGGGAAGAGACATTCGACGATGCGTACATCAAGCTGTATGAACTTTGGAAATCGAGAGGTCAATGACTATGTCAAACGTAAATACAGGTTCACTTAGTGTGGAc                                                                                                                                                                                                                                                                          <  1:29490/292‑1 (MQ=255)`  
                                    `aaTCTTAAAGTACAGACTAAGTGCTGGTAAGAAGTCAGAGTTAGCGTACTTAGAGAAAGACCTAGAGAAAGCAGACTTATATAAAGAACTCTTTGAGAAACATAAGGATAAATGTTATGCATAACTTCAAGTCAA‑CCCCACCTGCCGACAGCCTATCTGATGACTTCACATCTTGCTCAGAGTGGTGCCGAAAGATGTGGGAAGAGACATTCGACGATGCGTACATCAAGCTGTATGAACTTTGGAAATCGAGAGGTCAATGACTATGTCAAACGTAAATACAGGTTCACTTAGTGTGGAc                                                                                                                                                                                                                                                                          <  1:261641/300‑1 (MQ=255)`  
                                     `aTCTTAAAGTACAGACTACGTGCTGGTAAGAAGTCAGAGTTAGCGTACTTAGAGAAAGACCTAGCGAAAGCAGACTTCTATAAAGAACTCTTTGAGAAACATAAGGATAAATGTTATGCATAACTTCAAGTCAA‑CCCCACCTGCCGACAGCCTATCTGATGACTTCACATCTTGCTCAGAGTGGTCCCTAAAGATGTGGGAAGAGACATTCGACGATGCGTACATCAATCTGTATGAACTTTGGAAATCGAGAGGTCAATGACTATGTCAAACTTAAAtctgtctcttatacacatctccg                                                                                                                                                                                                                                                                         >  1:316426/1‑279 (MQ=255)`  
                                       `cTTAAAGTACAGACTACGTGCTGGTAAGAAGTCAGAGTTAGCGTACTTAGAGAAAGACCTAGCGAAAGCAGACTTCTATAAAGAACTCTTTGAGAAACATAAGGATAAATGTTATGCATAACTTCAAGTCAA‑CCCCACCTGCCGACAGCCTATCTGATGACTTCACATCTTGCTCAGAGTGGTGCCGAAAGATGTGGGAAGAGACATTCGACGATGCGTACATCAAGCTGTATGAACTTTGGAAATCGAGAGGTCAATGACTATGTCAAACGTAAATACAGGTTCACTTAGTGTGGACAAt                                                                                                                                                                                                                                                                       >  1:221977/1‑301 (MQ=255)`  
                                        `ttAAAGTACAGACTACGTGCTGGTAAGAAGTCAGAGTTAGCGTACTTAGAGAAAGACCTAGCGAAAGCAGACTTCTATAAAGAACTCTTTGAGAAACATAAGGATAAATGTTATGCATAACTTCAAGTCAA‑CCCCACCTGCCGACAGCCTATCTGATGACTTCACATCTTGCTCAGAGTGGTGCCGAAAGATGTGGGAAGAGACATTCGACGATGCGTACATCAAGCTGTATGAACTTTGGAAATCGAGAGGTCAATGACTATGTCAAACGTAAATACAGGTTCACTTAGTGTGGACAATa                                                                                                                                                                                                                                                                      >  1:156778/1‑301 (MQ=255)`  
                                           `aggTACAGACTACGTGCTGGTAAGAAGTCAGAGTTAGCGTACTTAGAGAAAGACCTAGCGAAAGCAGACTTCTATAAAGAACTCTTTGAGAAACATAAGGATAAATGTTATGCATAACTTCAAGTCAA‑CCCCACCTGCCGACAGCCTATCTGATGACTTCACATCTTGCTCAGAGTGGTGCCGAAAGATGTGGGAAGAGACATTCGACGATGCGTACATCAAGCTGTATGAACTCTGGAAATCGAGAGGTCAATGACTATGTCAAACGTAAATACAGGTTCACTTAGTGTGGACAATaaga                                                                                                                                                                                                                                                                   <  1:252798/299‑1 (MQ=255)`  
                                             `gTACAGACTACGTGCTGGTAAGAAGTCAGAGTTAGCGTACTTAGAGAAAGACCTAGCGAAAGCAGACTTCTATAAAGAACTCTTTGAGAAACATAAGGATAAATGTTATGCATAACTTCAAGTCAA‑CCCCACCTGCCGACAGCCTATCTGATGACTTCACATCTTGCTCAGAGTGGTGCCGAAAGATGTGGGAAGAGACATTCGACGATGCGTACATCATGCTGTATGAACTTTGGAAATCGAGAGGTCAATGACTATGTCAAACGTAAATACAGGTTCACTTAGTGTGGACAATaagctt                                                                                                                                                                                                                                                                 >  1:36119/1‑298 (MQ=255)`  
                                                  `gACTACGTGCTGGTAAGAAGTCAGAGTTAGCGTACTTAGAGAAAGACCTAGCGAAAGCAGACTTCTATAAAGAACTCTTTGAGAAACATAAGGATAAATGTTATGCATAACTTCAAGTCAA‑CCCCACCTGCCGACAGCCTATCTGATGACTTCACATCTTGCTCAGAGTGGTGCCGAAAGATGTGGGAAGAGACATTCGACGATGCGTACATCATGCTGTATGAACTTTGGAAATCGAGAGGTCAATGACTATGTCAAACGTAAATACAGGTTCACTTCGTGTGGAcctgtctcttataca                                                                                                                                                                                                                                                            >  1:120559/1‑287 (MQ=255)`  
                                                  `gACTACGTGCTGGTAAGAAGTCAGAGTTAGCGTACTTAGAGAAAGACCTAGCGAAAGCAGACTTCTATAAAGAACTCTTTGAGAAACATAAGGATAAATGTTATGCATAACTTCAAGTCAA‑CCCCACCTGCCGACAGCCTATCTGATGACTTCACATCTTGCTCAGAGTGGTGCCGAAAGATGTGGGAAGAGACATTCGACGATGCGTACATCAAGCTGTATGAACTTTGGAAATCGAGAGGTCAATGACTATGTCAAACGTAAATACAGGTTCACTTAGTGTGGACAATAAGAAGTTTTg                                                                                                                                                                                                                                                            >  1:5912/1‑301 (MQ=255)`  
                                                   `aCTACGTGCTGGTAAGAAGTCAGAGTTATCGTACTTAGAGAAAGACCTAGCGAAAGCAGACTTCTATAAAGAACTCTTTGAGAAACATAAGGATAAATGTTATGCATAACTTCAAGTCAACCCCCACCTGCCGACAGCCTATCTGATGACTTCACATCTTGCTCAGAGTGGTGCCGAAAGATGTGGGAAGAGACATTCGACGATGCGTACATCAAGCTGTATGAACTTTGGAAATCGAGAGGTCAATGACTATGTCAAACGTAAATACAGGTTCACTTAGTGTGGACAATAAGAATTTTTg                                                                                                                                                                                                                                                            >  1:183779/1‑301 (MQ=255)`  
                                                           `cTGGTAAGAAGTCAGAGTTAGAGTACTTAGAGAAAGACCTAGCGAAAGCAGACTTCTATAAAGAACTCTTTGAGAAACATAAGGATAAATGTTATGCATAACTTCAAGTCAA‑CCCCACCTGCCGACAGCCTATCTGATGACTTCACATCTTGCTCAGAGTGGTGCCGAAAGATGTGGGAAGAGACATTCGACGATGCGTACATCAAGCTGTATGAACTTTGGAAATCGAGAGGTGAATGACTATGTCAAACGTAAATACAGGTTCACTTAGTGTGGACAATAAGAAGTTTTGGGCTACCGt                                                                                                                                                                                                                                                   <  1:297546/301‑1 (MQ=255)`  
                                                             `ggTAAGAAGTCAGAGTTAGCGTACTTAGAGAAAGACCTAGCGAAAGCAGACTTCTATAAAGAACTCTTTGAGAAACATAAGGATAAATGTTATGCATAACTTCAAGTCAA‑CCCCACCTGCCGACAGCCTATCTGATGACTTCACATCTTGCTCAGAGTGGTGCCGAAAGATGTGGGAAGAGACATTCGACGATGCGTACATCAAGCTGTATGAACTTTGGAAATCGAGAGGTCAATGACTATGTCAAACGTAAATACAGGTTCACTTAGTGTGGACAATAAGAAGTTTTGGGCTACCGTag                                                                                                                                                                                                                                                 <  1:285082/301‑1 (MQ=255)`  
                                                               `tAAGAAGTCAGAGTTAGCGTACTTAGAGAAAGACCTAGCGAAAGCAGACTTCTATAAAGAACTCTTTGAGAAACATAAGGATAAATGTTATGCATAACTTCAAGTCAA‑CCCCACCTGCCGACAGCCTATCTGATGACTTCACATCTTGCTCAGAGTGGTGCCGAAAGATGTGGGAAGAGACATTCGACGATGCGTACATCACGCTGTATGAACTTTGGAAATCGAGAGGTCAATGACTATGTCAAACGTAAATACAGGTTCACTTAGTGTGGACAATacgactgtctcttatacacatctc                                                                                                                                                                                                                                               >  1:197787/1‑281 (MQ=255)`  
                                                                       `tggggtcggagatgtgtataagagacagaGCGAAAGCAGACTTCTATAAAGAACTCTTTGAGAAACATAAGGATAAATGTTATGCATAACTTCAAGTCAA‑CCCCACCTGCCGACAGCCTATCTGATGACTTCACATCTTGCTCAGAGTGGTGCCGAAAGATGTGGGAAGAGACATTCGACGATGCGTACATCAAGCTGTATGAACTTTGGAAATCGAGAGGTCAATGACTATGTCAAACGTAAATACAGGTTCACTTAGTGTGGACAATAAGAAGTTTTGGGCTACCGTAGAGTCCTCGGa                                                                                                                                                                                                                                       <  1:142434/273‑1 (MQ=255)`  
                                                                         `gagTTAGCATACTTAGAGAAAGACCTAGCGAAAGCAGACTTCTATAAAGAACGCTTTGAGAAACATAAGGAAAAAGGTTATGAATAACTTCAAGTCAA‑CCCCACCTGCCGACAGCCTATCTGATGACTTCACATCTTGCTCAGAGTGGTGCCGGAAGATGTGGGAAGAGACATTCGACGATGCGTACATCAAGCTGTATGAACTTTGGAAATCGAGAGGTCAATGACTATGTCAAACGTAAATAGAGGTTCACTTAGTGTGGACAATAAGAAGTTTTGGGCTACCGTAGAGTCCTCGGAGc                                                                                                                                                                                                                                     <  1:66897/301‑1 (MQ=255)`  
                                                                                `agTACTTAGAGAAAGACCTAGCGAAAGCAGACTTCTATAAAGAACTCTTTGAGAAACATAAGGATAAATGTTATGCATAACTTCAAGTCAA‑CCCCACCTGCCGACAGCCTATCTGATGACTTCACATCTTGCTCAGAGTGGTGCCGAAAGATGTGGGAAGAGACATTCGACGATGCGTACATCAAGCTGTATGAACTTTGGAAATCGAGAGGTCAATGACTATGTCAAACGTAAATACAGGTTCACTTAGTGTGGACAATAAGAAGTTTTGGGCTACCGTAGAGTCCTCGGAGCATTCCtt                                                                                                                                                                                                                              <  1:5361/300‑1 (MQ=255)`  
                                                                                        `gagaAAGACCTAGCGAAAGCAGACTTCTATAAAGAACTCTTTGAGAAACATAAGGATAAATGTTATGCATAACTTCAAGTCAA‑CCCCACCTGCCGACAGCCTATCTGATGACTTCACATCTTGCTCAGAGTGGTGCCGAAAGATGTGGGAAGAGACATTCGACGATGCGTACATCAAGCTGTATGAACTTTGGAAATCGAGAGGTCAATGACTATGTCAAACGTAAATACAGGTTCACTTAGTGTGGACAATAAGAAGTTTTGGGCTACCGTAGAGTCCTCGGAGCATTCATTCGAGGTTc                                                                                                                                                                                                                      >  1:82439/1‑301 (MQ=255)`  
                                                                                             `aGACCTAGCGAAAGCAGACTTCTATAAAGAACTCTTTGAGAAACATAAGGATAAATGTTATGCATAACTTCAAGTCAA‑CCCCACCTGCCGACAGCCTATCTGATGACTTCACATCTTGCTCAGAGTGGTGCCGAAAGATGTGGGAAGAGACATTCGACGATGCGTACATCAAGCTGTATGAACTTTGGAAATCGAGAGGTCAATGACTATGTCAAACGTAAATACAGGTTCACTTAGTGTGGACAATAAGAAGTTTTGGGCTACCGTAGAGTCCTCGGAGCATTCCTTCGAGGTTCCAATc                                                                                                                                                                                                                 <  1:72588/301‑1 (MQ=255)`  
                                                                                             `aGACCTAGCGAAAGCAGACTTCTATAAAGAACTCTTTGAGAAACATAAGGATAAATGTTATGCATAACTTCAAGTCAA‑CCCCACCTGCCGACAGCCTATCTGATGACTTCACATCTTGCTCAGAGTGGTGCCGAAAGATGTGGGAAGAGACATTCGACGATGCGTACATCAAGCTGTATGAACTTTGGAAATCGAGAGGTCAATGACTATGTCAAACGTAAATACAGGTTCACTTAGTGTGGACAATAAGAAGTTTTGGGCTACCGTAGAGTCCTCGGAGCATTCCTTCGAGGTTCCAATc                                                                                                                                                                                                                 <  1:206405/301‑1 (MQ=255)`  
                                                                                             `aGACCTAGCGAAAGCAGACTTCTATAAAGAACTCTTTGAGAAACATAAGGATAAATGTTATGCATAACTTCAAGTCAA‑CCCCACCTGCCGACAGCCTATCTGATGACTTCACATCTTGCTCAGAGTGGTGCCGAAAGATGTGGGAAGAGACATTCGACGATGCGTACATCAAGCTGTATGAACTTTGGAAATCGAGAGGTCAATGACTATGTCAAACGTAAATACAGGTTCACTTAGTGTGGACAATAAGAAGTTTTGGGCTACCGTAGAGTCCTCGGAGCATTCCTTCGAGGTTCCAATc                                                                                                                                                                                                                 <  1:216293/301‑1 (MQ=255)`  
                                                                                             `aGACCTAGCGAAAGCAGACTTCTATAAAGAACTCTTTGAGAAACATAAGGATAAATGTTATGCATAACTTCAAGTCAA‑CCCCACCTGCCGACAGCCTATCTGATGACTTCACATCTTGCTCAGAGTGGTGCCGAAAGATGTGGGAAGACACATTCGACGATGCGTACATCAAGCTGTATGAACTTTGGAAATCGAGAGGTCAATGACTATGTCAAACGTAAATACAGGTTCACTTAGTGTGGACAATAAGAAGTTTTGGGCTACCGTAGAGTCCTCGGAGCATTCCTTCGAGGTTCCAATc                                                                                                                                                                                                                 <  1:112076/301‑1 (MQ=255)`  
                                                                                              `gACCTAGCGAAAGCAGACTTCTATAAAGAACTCTTTGAGAAACATAAGGATAAATGTTATGCATAACTTCAAGTCAA‑CCCCACCTGCCGACAGCCTATCTGATGACTTCACATCTTGCTCAGAGTGGTGCCGAAAGATGTGGGAAGAGACATTCGACGATGCGTACATCAAGCTGTATGAACTTTGGAAATCGAGAGGTCAATGACTATGTCAAACGTAAATACAGGTTCACTTAGTGTGGACAATAAGAAGTTTTGGGCTACCGTAGAGTCCTCGGAGCATTCCTTCGAGGTTCCAATCt                                                                                                                                                                                                                <  1:154046/301‑1 (MQ=255)`  
                                                                                              `aaCCTAGCGAAAGCAGACTTCTATAAAGAACTCTTTGAGAAACATAAGGATAAATGTTATGCATAACTTCAAGTCAA‑CCCCACCTGCCGACAGCCTATCTGATGACTTCACATCTTGCTCAGAGTGGTGCCGAAAGATGTGGGAAGAGACATTCGACGATGCGTACATCAAGCTGTATGAACTTTGGAAATCGAGAGGTCAATGACTATGTCAAACGTAAATACAGGTTCACTTAGTGTGGACAATAAGAAGTTATGGGCTACCGTAGAGTCCTCGGAGCATTCCTTCGAGGTTCCAATCt                                                                                                                                                                                                                <  1:299168/300‑1 (MQ=255)`  
                                                                                                `ccTAGCGAAAGCAGACTTCTATAAAGAACTCTTTGAGAAACATAAGGATAAATGTTATGCATAACTTCAAGTCAA‑CCCCACCTGCCGACAGCCTATCTGATGACTTCACATCTTGCTCAGAGTGGTGCCGAAAGATGTGGGAAGAGACATTCGACGATGCGTACATCAAGCTGTATGAACTTTGGAAATCGAGAGGTCAATGACTATGTCAAACGTAAATACAGGTTCACTTAGTGTGGACAATAAGAAGTTTTGGGATACCGTAGAGTCCTCGGAGCATTCCTTCGAGGTTCCAATCTAc                                                                                                                                                                                                              >  1:302077/1‑301 (MQ=255)`  
                                                                                                   `aGCGAAAGCAGACTTCTATAAAGAACTCTTTGAGAAACATAAGGATAAATGTTATGCATAACTTCAAGTCAA‑CCCCACCTGCCGACAGACTATCTGATGACTTCACATCTTGCTCAGAGTGGTGCCGAAAGATGTGGGAAGAGACATTCGACGATGCGTACATCAAGCTGTATGAACTTTGGAAATCGAGAGGTCAATGACTATGTCAAACGTAAATACAGGTTCACTTAGTGTGGACAATAAGAAGTTTTGGGCTACCGTAGAGTCCTCGGAGCATTCCTTCGAGGTTCCAATCTACGCt                                                                                                                                                                                                           <  1:97589/301‑1 (MQ=255)`  
                                                                                                     `cGAAAGCAGACTTCTATAAAAAACTCTTGGATAAACAGCAGGATAAATGTTATGCATAACTTCAAGTCAA‑CCCCACCTGCCGACAGCCTATCTGATGACTTCACATCTTGCTCAGAGTGGTGCCGAAAGATGTGGGGAGAGACATTCGACGATGCGTACATCAAGCTGTAGGAACTGTGGAAATCGAGAGGTCAATGACTATGTCAAACGTAAATACAGGTTCACTTAGTGTGGACAATAAGAAGTTTTGGGCTACCGTAGAGTCCTCGGAGCATTCCTTCGAGGTTCCAATCTACGCTga                                                                                                                                                                                                         <  1:159288/301‑1 (MQ=255)`  
                                                                                                              `gagatgtgtataagagacaggagaAACATAAGGATAAATGTTATGCATAACTTCAAGTCAA‑CCCCACCTGCCGACAGCCTATCTGATGACTTCACATCTTGCTCAGAGTGGTGCCGAAAGATGTGGGAAGAGACATTCGACGATGCGTACATCAAGCTGTATGAACTTTGGAAATCGAGAGGTCAATGACTATGTCAAACGTAAATACAGGTTCACTTAGTGTGGACAATAAGAAGTTTTGGGCTACCGTAGAGTCCTCGGAGCATTCCTTCGAGGTTCCAATCTACGCTGAGACCCTAGa                                                                                                                                                                                                <  1:39802/281‑1 (MQ=255)`  
                                                                                                               `cTTCTATACAGAACTCTTTGAGAAACATAAGGATAAATGTTATGCATAACTTCAAGTCAA‑CCCCACCTGCCGACAGCCTATCTGATGACTTCACATCTTGCTCAGAGTGGTGCCGAAAGATGTGGGAAGAGACATTCAACGATGCGTACATCAAGCTGTATGAACTTTGGAAATCGAGAGGTCAATGACTATGTCAAACGTAAATACAGGTTCACTTAGTGTGGACAATAAGAAGTTTTGGGCTACCGTAGAGTCCTCGGAGCATTCCTTCGAGGTTCCAATCTACGCTGAGACCCTAGAc                                                                                                                                                                                               >  1:145833/1‑301 (MQ=255)`  
                                                                                                               `cTTCTATAAAAAACACTTTAAGAAACATAAGGATAAATGTTATGCATAACTTCAAGTCAA‑CCCCACCTGCCGACAGCCGAGCTGATGACTTCACATCTTGCTCAGAGTGGTGCCGAAAGATGTGGGAAGAGACATTCGACGATGCGTACATCAAGCTGTATGAACTTTGGAAATCGAGAGGTCAATGACTATGTCAAACGTAAATACAGGTTCACTTAGTGTGGACAATAAGAAGTTTTGGGATACCGTAGAGTCCTCGGAGCATTCCTTCGAGGTTCCAATCTACGCTGAGACCCTAGAc                                                                                                                                                                                               <  1:331871/301‑1 (MQ=255)`  
                                                                                                                 `tCTATAAAGAACTCTTTGAGAAACATAAGGATAAATGTTATGCATAACTTCAAGTCAA‑CCCCACCTGCCGACAGCCTATCTGATGACTTCACATCTTGCTCAGAGTGGTGCCGAAAGATGTGGGAAGAGACATTCGACGATGCGTACATCAAGCTGTATGAACTTTGGAAATCGAGAGGTCAATGACTATGTCAAACGTAAATACAGGTTCACTTAGTGTGGACAATAAGAAGTTTTGGGCTACCGTAGAGTCCTCGGAGCATTCCTTCGAGGTTCCAATCTACGCTGAGAcccctgtctc                                                                                                                                                                                             >  1:194893/1‑294 (MQ=255)`  
                                                                                                                  `cTATAAAGAACTCTTTGAGAAACATAAGGATAAATGTTATGCATAACTTCAAGTCAA‑CCTCACCTGCCGACAGTCTATCTGATGACTTCACATCTTGCTCAGAGTGGTGCCGAAAGATGTGGGTAGAGACATTCGACGATGCGTACATCAAGCTGTATGAACTTTGGAAATCGAGAGGTCAATGACTATGTCAAACGTAAATACAGGTTCACTTAGTGTGGACAATAAGAAGTTTTGGGCTACCGTAGAGTCCTCGGAGCATTCCTTCGAGGTTCCAATCTACGCTGAGACCCTAGACGaa                                                                                                                                                                                            >  1:116951/1‑301 (MQ=255)`  
                                                                                                                    `ataAAGAACTCTTTGAGAAACATAAGGATAAATGTTATGCATAACTTCAAGTCAA‑CCCCACCTGCCGACAGCCTATCTGATGACTTCACATCTTGCTCAGAGTGGTGCCGAAAGATGTGGGAAGAGACATTCGACGATGCGTACATCAAGCTGTATGAACTTTGGAAATCGAGAGGTCAATGACTATGTCAAACGTAAATACAGGTTCACTTAGTGTGGACAATAAGAAGTTTTGGGCTACCGTAGAGTCCTCGGAGCATTCCTTCGAGGTTCCAATCTACGCTGAGACCCTAGACGAAGc                                                                                                                                                                                          >  1:170793/1‑301 (MQ=255)`  
                                                                                                                    `ataAAGAACTCTTTGAGAAACATAAGGATAAATGTTATGCATAACTTCAAGTCAA‑CCCCACCTGCCGACAGCCTATCTGATGACTTCACATCTTGCTCAGAGTGGTGCCGAAAGATGTGGGAAGAGACATTCGACGATGCGTACATCAAGCTGTATGAACTTTGGAAATCGAGAGGTCAATGACTATGTCAAACGTAAATACAGGTTCACTTAGTGTGGACAATAAGAAGTTTTGGGCTACCGTAGAGTCCTCGGAGCATTCCTTCGAGGTTCCAATCTACGCTGAGACCCTAGACGAAGc                                                                                                                                                                                          >  1:264192/1‑301 (MQ=255)`  
                                                                                                                         `gAACTCTTTGAGAAACATAAGGATAAATGTTATGCATAACTTCAAGTCAA‑CCCCACCTGCCGACAGCCTATCTGATGACTTCACATCTTGCTCAGAGTGGTGCCGAAAGATGTGGGAAGAGACATTCGACGATGCGTACATCAAGCTGTATGAACTTTGGAAATCGAGAGGTCAATGACTCTGTCAAACGTAAATACAGGTTCACTTAGTGTGGACAATAAGAAGTTTTGGGCTACCGTAGAGTCCTCGGAGCATTCCTTCGAGGTTCCAATCTACGCTGAGACCCTAGACGAAGCTCTgg                                                                                                                                                                                     >  1:104056/1‑301 (MQ=255)`  
                                                                                                                           `aCTCTTTGAGAAACATAAGGATAAATGTTATGCATAACTTCAAGTCAA‑CCCCACCTGCCGACAGCCTATCTGATGACTTCACATCTTGCTCAGAGTGGTGCCGAAAGATGTGGGAAGAGACATTCGACGATGCGGACATCAAGCTGTATGAACTTTGGAAATCGAGAGGTCAATGACTATGTCAAACGTAAATACAGGTTCACTTAGTGTGGACAATAAGAAGTTTTGGGCTACCGTAGAGTCCTCGGAGCATTCCTTCGAGGTTCCAATCTACGCTGAGACCCTAGACGAAGCTCTGGAg                                                                                                                                                                                   <  1:267933/301‑1 (MQ=255)`  
                                                                                                                                            `aGGATAAATGTTATGCATAACTTCAAGTCAA‑CCCCACCTGCCGACAGCCTATCTGATGACTTCACATCTTGCTCAGAGTGGTGCCGAAAGATGTGGGAAGAGACATTCGACGATGCGTACATCAAGCTGTATGAACTTTGGAAATCGAGAGGTCAATGACTATGTCAAACGTAAATACAGGTTCACTTAGTGTGGACAATAAGAAGTTTTGGGCTACCGTAGAGTCCTCGGAGCATTCCTTCGAGGTTCCACTCTACGCTGAGACCCTAGACGAAGCTCTGGAGTTAGCCGAATGGCAATa                                                                                                                                                                  >  1:95050/1‑301 (MQ=255)`  
                                                                                                                                             `ggATAAATGTTATGCATAACGTCAAGTCAA‑CCCCACCTGCCGACAGCCTATCTGATGACTTCACATCTTGCTCAGAGTGGTGCCGAAAGATGTGGGAAGAGACATTCGACGATGCGTACATCAAGCTGTATGAACTTTGGAAATCGAGAGGTCAATGACTATGTCAAACGTAAATACAGGTTCACTTAGTGTGGACAATAAGAAGTTTTGGGCTACCGTAGAGTCCTCGGAGCATTCCTTCGAGGTTCCAATCTACGCTGAGACCCTAGACGAAGCTCTGGAGTTAGCCGAATGGCAATAc                                                                                                                                                                 <  1:85129/301‑1 (MQ=255)`  
                                                                                                                                              `gATAAATGTTATGCATAACTTCAAGTCAA‑CCCCACCTGCCGACAGCCTATCTGATGACTTCACATCTTGCTCAGAGTGGTGCCGAAAGATGTGGGAAGAGACATTCGACGATGCGTACATCAAGCTGTATGAACTTTGGAAATCGAGAGGTCAATGACTATGTCAAACGTAAATACAGGTTCACTTAGTGTGGACAATAAGAAGTTTTGGGCTACCGTAGAGTCCTCGGAGCATTCCTTCGAGGTTCCAATCTACGCTGAGACCCTAGACGAAGCTCTGGAGTTAGCCGAATGGCAATACg                                                                                                                                                                >  1:256195/1‑301 (MQ=255)`  
                                                                                                                                               `aTAAATGTTATGCATAACTTCAAGTCAA‑CCCAACCTGCCGACAGCCTATCTGATGACTTCACATCTTGCTCAGAGTGGTGCCGAAAGATGTGGGAAGAGACATTCGACGATGCGTACATCAAGCTGTATGAACTTTGGAAATCGAGAGGTCAATGACTATGTCAAACGTAAATACAGGTTCACTTAGTGTGGACAATAAGAAGTTTTGGGCTACCGTAGAGTCCTCGGAGCATTCCTTCGAGGTTCCAATCTACGCTGAGACCCTAGACGAAGCTCTGGAGTTAGCCGAATGGCAATACGt                                                                                                                                                               >  1:340536/1‑301 (MQ=255)`  
                                                                                                                                                   `aTGTTATGCATAACTTCAAGTCAA‑CCCCACCTGCCGACAGCCTATCTGATGACTTCACATCTTGCTCAGAGTGGTGCCGAAAGATGTGGGAAGAGACATTCGACGATGCGTACATCAAGCTGTATGAACTTTGGAAATCGAGAGGTCAATGACTATGTCAAACGTAAATACAGGTTCACTTAGTGTGGACAATAAGAAGTTTTGGGCTACCGTAGAGTCCTCGGAGCATTCCTTCGAGGTTCCAATCTACGCTGAGACCCTAGACGAAGCTCTGGAGTTAGCCGAATGGCGATACGTTCCg                                                                                                                                                           >  1:277945/1‑301 (MQ=255)`  
                                                                                                                                                     `gTTATGCATAACTTCAAGTCAA‑CCCCACCTGCCGACAGCCTATCTGATGACTTCACATCTTGCTCAGAGTGGTGCCGAAAGATGTGGGAAGAGACATTCGACGATGCGTACATCAAGCTGTATGAACTTTGGAAATCGAGAGGTCAATGACTATGTCAAACGTAAATACAGGTTCACTTAGTGTGGACAATAAGAAGTTTTGGGCTACCGTAGAGTCCTCGGAGCATTCCTTCGAGGTTCCAATCTACGCTGAGACCCTAGACGAAGCTCTGGAGTTAGCCGAATGGCAATACGTTCCggc                                                                                                                                                         >  1:219087/1‑301 (MQ=255)`  
                                                                                                                                                        `gctcggagaggtgtataagagacagccTGCCGACAGCCTATCTGATGACTTCACATCTTGCTCAGAGTGGTGCCGAAAGATGTGGGAAGAGACATTCGACGATGCGTACATCAAGCTGTATGAACTTTGGAAATCGAGAGGTCAATGACTATGTCAAACGTAAATACAGGTTCACTTAGTGTGGACAATAAGAAGTTTTGGGCTACCGTAGAGTCCTCGGAGCATTCCTTCGAGGTTCCAATCTACGCTGAGACCCTAGACGAAGCTCTGGAGTTAGCCGAATGGCAATACGTTCCggctg                                                                                                                                                       <  1:177021/276‑1 (MQ=255)`  
                                                                                                                                                          `gCATAACTTCAAGTCAA‑CCCCACCTGCCGACAGCCTATCTGATGACTTCACATCTTGCTCAGAGTGGTGCCGAAAGATGTGGGAAGAGACATTCGACGATGCGTACATCAAGCTGTATGAACTTTGGAAATCGAGAGGTCAATGACTATGTCAAACGTAAATACAGGTTCACTTAGTGTGGACAATAAGAAGTTTTGGGCTACCGTAGAGTCCTCGGAGCATTCCTTCGAGGTTCCAATCTACGCTGAGACCCTAGACGAAGCTCTGGAGTTAGCCGAATGGCAATACGTTCCggctggct                                                                                                                                                    >  1:168047/1‑301 (MQ=255)`  
                                                                                                                                                          `gCATAACTTCAAGTCAA‑CCCCACCTGCCGACAGCCTATCTGATGACTTCACATCTTGCTCAGAGTGGTGCCGAAAGATGTGGGAAGAGACATTCGACGATGCGTACATCAAGCTGTATGAACTTTGGAAATCGAGAGGTCAATGACTATGTCAAACGTAAATACAGGTTCACTTAGTGTGGACAATAAGAAGTTTTGGGCTACCGTAGAGTCCTCGGAGCATTACTTCGAGGTTCCAATCTACGCTGAGACCCTAGACGAAGCTCTGGAGTTAGCCGAATGGCAATACGTTCCggctggct                                                                                                                                                    >  1:273722/1‑301 (MQ=255)`  
                                                                                                                                                                `cTTCAAGTCAA‑CCCCACCTGCCGACAGCCTATCTGATGACTTCACATCTTGCTCAGAGTGGTGCCGAAAGATGTGGGAAGAGACATTCGACGATGCGTACATCAAGCTGTATGAACTTTGGAAATCGAGAGGTCAATGACTATGTCAAACGTAAATACAGGTTCACTTAGTGTGGACAATAAGAAGTTTTGGGCTACCGTAGAGTCCTCGGAGCATTCCTTCGAGGTTCCAATCTACGCTGAGACCCTAGACGAAGCTCTGGAGTTAGCCGAATGGCAATACGTTCCGGCTGGCTTTGAgg                                                                                                                                              >  1:231126/1‑301 (MQ=255)`  
                                                                                                                                                                `cTTCAAGTCAA‑CCCCACCTGCCGACAGCCTATCTGATGACTTCACATCTTGCTCAGAGTGGTGCCGAAAGATGTGGGAAGAGACATTCGACGATGCGTACATCAAGCTGTATGAACTTTGGAAATCGAGAGGTCAATGACTATGTCAAACGTAAATACAGGTTCACTTAGTGTGGACAATAAGAAGTTTTGGGCTACCGTAGAGTCCTCGGAGCATTCCTTCGAGGTTCCAATCTACGCTGAGACCCTAGACGAAGCTCTGGAGTTAGCCGAATGGCAATACGTTCCGGCTGGCTTTGAgg                                                                                                                                              >  1:240905/1‑301 (MQ=255)`  
                                                                                                                                                                     `aGTCAA‑CCCCACCTGCCGACAGCCTATCTGATGACTTCACATCTTGCTCAGAGTGGTGCCGAAAGATGTGGGAAGAGACATTCGACGATGCGTACATCAAGCTGTATGAACTTTGGAAATCGAGAGGTCAATGACTATGTCAAACGTAAATACAGGTTCACTTAGTGTGGACAATAAGAAGTTTTGGGCTACCGTAGAGTCCTCGGAGCATTCCTTCGAGGTTCCAATCTACGCTGAGACCCTAGACGAAGCTCTGGAGTTAGCCGAATGGCAATACGTTCCGGCTGGCTTTGAGGTTACt                                                                                                                                         <  1:235111/301‑1 (MQ=255)`  
                                                                                                                                                                         `aa‑CCCCACCTGCCGACAGCCTATCTGATGACTTCACATCTTGCTCAGAGTGGTGCCGAAAGATGTGGGAAGAGACATTCGACGATGCGTACATCAAGCTGTATGAACTTTGGAAATCGAGAGGTCAATGACTATGTCAAACGTAAATACAGGTTCACTTAGTGTGGACAATAAGAAGTTTTGGGCTACCGTAGAGTCCTCGGAGCATTCCTTCGAGGTTCCAATCTACGCTGAGACCCTAGACGAAGCTCTGGAGTTAGCCGAATGGCAATACGTTCCGGCTGGCTTTGAGGTTACTCgtg                                                                                                                                     >  1:929/1‑301 (MQ=255)`  
                                                                                                                                                                         `aa‑CCCCACCTGCCGACAGCCTATCTGATGACTTCACATCTTGCTCAGAGTGGTGCCGAAAGATGTGGGAAGAGACATTCGACGATGCGTACATCAAGCTGTATGAACTTTGGAAATCGAGAGGTCAATGACTATGTCAAACGTAAATACAGGTTCACTTAGTGTGGACAATAAGAAGTTTTGGGCTACCGTAGAGTCCTCGGAGCATTCCTTCGAGGTTCCAATCTACGCTGAGACCCTAGACGAAGCTCTGGAGTTAGCCGAATGGCAATACGTTCCGGCTGGCTTTGAGGTTACTCgtg                                                                                                                                     <  1:69738/301‑1 (MQ=255)`  
                                                                                                                                                                            `ccccACCTGCCGACAGCCTATCTGATGACTTCACATCTTGCTCAGAGTGGTGCCGAAAGATGTGGGAAGAGACATTCGACGATGCGTACATCAAGCTGTATGAACTTTGGAAATCGAGAGGTCAATGACTATGTCAAACGTAAATACAGGTTCACTTAGTGTGGACAATAAGAAGTTTTGGGCTACCGTAGAGTCCTCGGAGCATTCCTTCGAGGTTCCAATCTACGCTGAGACCCTAGACGAAGATCTGGAGTTAGCCGAATGGCAATACGTTCCGGCTGGCTTTGAGGTTACTCGtgtg                                                                                                                                   <  1:106548/301‑1 (MQ=255)`  
                                                                                                                                                                                       `gACAGCCTATCTGATTACTTCACATCTTGCTCAGAGTGGTGCCGAAAGATGTGGGAAGAGACATTCGACGATGCGTACATCAAGCTGTATGAACTTTGGAAATCGAGAGGTCAATGACTATGTCAAACGTAAATACAGGTTCACTTAGTGTGGACAATAAGAAGTTTTGGGCTACCGTAGAGTCCTCGGAGCATTCCTTCGAGGTTCCAATCTACGCTGAGACCCTAGACGAAGCTCTGGAGTTAGCCGAATGGCAATACGTTCCGGCTGGCTTTGAGGTTACTCGTGTGCGTCCTTgtgt                                                                                                                        <  1:312379/301‑1 (MQ=255)`  
                                                                                                                                                                                       `gACAGCCTATCTGATGACTTCACATCTTGCTCAGAGTGGTGCCGAAAGATGTGGGAAGAGACATTCGACGATGCGTACATCAAGCTGTATGAACTTTGGAAATCGAGAGGTCAATGACTATGTCAAACGTAAATACAGGTTCACTTAGTGTGGACAATAAGAAGTTTTGGGCTACCGTAGAGTCCTCGGAGCATTCCTTCGAGGTTCCAATCTACGCTGAGACCCTAGACGAAGCTCTGGAGTTAGCCGAATGGCAATACGTTCCGGCTGGCTTTGAGGTTACTCGTGTGCGGCCTTgtgt                                                                                                                        <  1:173995/301‑1 (MQ=255)`  
                                                                                                                                                                                        `aCAGCCTATCTGATGACTTCACATCTTGCTCAGAGTGGTGCCGAAAGATGTGGGAAGAGACATTCGACGATGCGTACATCAAGCTGTATGAACTTTGGAAATCGAGAGGTCAATGACTATGTCAAACGTAAATACAGGTTCACTTAGTGTGGACAATAAGAAGTTTTGGGCTACCGTAGAGTCCTCGGAGCATTCCTTCGAGGTTCCAATCTACGCTGAGACCCTAGACGAAGCCCTGGAGTTAGCCGAATGGCAATACGTTCCGGCTGGCTTTGAGGTTACTCGTGTGCGTCCTTgtgtg                                                                                                                       >  1:291604/1‑300 (MQ=255)`  
                                                                                                                                                                                         `aaGCCTATCTGATGACTTCACATCTTGCTCAGAGTGGTGCCGAAAGATGTGGGAAGAGACATTCGACGATGCGTACATCAAGCTGTATGAACTTTGGAAATCGAGAGGTCAATGACTATGTCAAACGTAAATACAGGTTCACTTAGTGTGGACAATAAGAAGTTTTGGGCTACCGTAGAGTCCTCGGAGCATTCCTTCGAGGTTCCAATCTACGCTGAGACCCTAGACGAAGCTCTGGAGTTAGCCGAATGGCAATACGTTCCGGCTGGCTTTGAGGTTACTCGTGTGCGTCCTTGTGTAg                                                                                                                      <  1:208629/300‑1 (MQ=255)`  
                                                                                                                                                                                         `aaGCCTATATGAAGACTTCACATCTTGCTCAGAGTGGTGCCGAAAGATGTAGGAAAAAACATACGAAAAAGCGAACAACAAGCTGTATGAACTATGGAAATCGAGAGGTCAATGACTATGTCAAACGTAAATACAGGTTCACTTAGTGTGGACAATAAGAAGTTTTGGGCTACCGTAGAGTCCTCGGAGCATTCCTTCGAGGTTCCAATCTACGCTGAAACCCTAGACGAAGCTCTGGAGTTAGCCGAATGGCAATACGTTCCGGCTGGCTTTGAGGTTACTCGTGTGCGTCCTTGTGTAg                                                                                                                      <  1:305510/300‑1 (MQ=255)`  
                                                                                                                                                                                                `tCTGATGACTTCACATCTTGCTCAGAGTGGTGCCGAAAGATGTGGGAAGAGACATTCGACGATGCGTACATCAAGCTGTATGAACTTTGGAAATCGAGAGGTCAATGACTATGTCAAACGTAAATACAGGTTCACTTAGTGTGGACAATAAGAAGTTTTGGGCTACCGTAGAGTCCTCGGAGCATTCCTTCGAGGTTCCAATCTACGCTGAGACCCTAGACGAAGCTCTGGAGTTAGCCGAATGGCAATACGTTCCGGCTGGCTTTGAGGTTACTCGTGTGCGTCCTTGTGTAGCACCGaa                                                                                                               >  1:114152/1‑301 (MQ=255)`  
                                                                                                                                                                                                 `tttgtgaCTTCACATCTTGGTCGGAGTGGGGCCGAAAGATGTGGGAAGAGACATTCGACGATGCGTACATCAAGCTGTATGAACTTTGGAAATCGAGAGGTCAATGACTATGTCAAACGTAAATACAGGTTCACTTAGTGTGGACAATAAGAAGTTTTGGGCTACCGTAGAGTCCTCGGAGCATTCCTTCGAGGTTCCAATCTACGCTGAGACCCTAGACGAAGCTCTGGAGTTAGCCGAATGGCAATACGTTCCGGCTGGCTTTGAGGTTACTCGTGTGCGTCCTTGTGTAGCACCGAAg                                                                                                              <  1:150468/297‑1 (MQ=255)`  
                                                                                                                                                                                                       `gagacagaTCTTGCTCAGAGTGGGGCCGAAAGATGTTGGAAGAGACATTCGACGATGCGTACATCAAGCTGTATGAACTTTGGAAATCGAGAGGTCAATGACTATGTCAAACGTAAATACAGGTTCACTTAGTGTGGACAATAAGAAGTTTTGGGCTACCGTAGAGTCCTCGGAGCATTCCTTCGAGGTTCCAATCTACGCTGAGACCCTAGACGAAGCTCTGGAGTTAGCCGAATGGCAATACGTTCCGGCTGGCTTTGAGGTTACTCGTGTGCGTCCTTGTGTAGCACCGAAGTAATAc                                                                                                        <  1:212778/294‑1 (MQ=255)`  
                                                                                                                                                                                                       `ataagagacagtGCTCAGAGTGGTGCCGAAAGATGTGGGAAGAGACATTCGACGATGCGTACATCAAGCTGTATGAACTTTGGAAATCGAGAGGTCAATGACTATGTCAAACGTAAATACAGGTTCACTTAGTGTGGACAATAAGAAGTTTTGGGCTACCGTAGAGTCCTCGGAGCATGCCTTCGAGGTTCCAATCTACGCTGAGACCCTAGACGAAGCTCTGGAGTTAGCCGAATGGCAATACGTTCCGGCTGGCTTTGAGGTTACTCGTGTGCGTCCTTGTGTAGCACCGAAGTAATAc                                                                                                        <  1:63176/290‑1 (MQ=255)`  
                                                                                                                                                                                                       `aCTTCACATCTTGCTCAGAGTGGTGCCGAAAGATGTGGGAAGAGACATTCGACGATGCGTACATCAAGCTGTATTAACTTTGGAAATCGAGAGGTCAATGACTATGTCAAACGTAAATACAGGTTCACTTAGTGTGGACAATAAGAAGTTTTGGGCTACCGTAGAGTCCTCGGAGCATTCCTTCGAGGTTCCAATCTACGCTGAGACCCTAGACGAAGCTCTGGAGTTAGCCGAATGGCAATACGTTCCGGCTGGCTTTGAGGTTACTCGTGTGCGTCCTTGTGTAGCACCGAAGTAATAc                                                                                                        <  1:245327/301‑1 (MQ=255)`  
                                                                                                                                                                                                       `aCTTCACATCTTGCTCAGAGTGGTGCCGAAAGATGTGGGAAGAGACATTCGACGATGCGTACATCAAGCTGTATGAACTTTGGAAATCGAGAGGTCAATGACTATGTCAAACGTAAATACAGGTTCACTTAGTGTGGACAATAAGAAGTTTTGGGCTACCGTAGAGTCCTCGGAGCATTCCTTCGAGGTTCCAATCTACGCTGAGACCCTAGACGAAGCTCTGGAGTTAGCCGAATGGCAATACGTTCCGGCTGGCTTTGAGGTTACTCGTGTGCGTCCTTGTGTAGCACCGAAGTAATAc                                                                                                        <  1:188459/301‑1 (MQ=255)`  
                                                                                                                                                                                                       `aCTTCACATCTTGCTCAGAGTGGTGCCGAAAGATGTGGGAAGAGACATTCGACGATGCGTACATCAAGCTGTATGAACTTTCGAAATCGAGAGGTCAATGACTATGTCAAACGTAAATACAGGTTCACTTAGTGTGGACAATAAGAAGTTTTGGGCTACCGTAGAGTCCTCGGAGCATTCCTTCGAGGTTCCAATCTACGCTGAGACCCTAGACGAAGCTCTGGAGTTAGCCGAATGGCAATACGTTCCGGCTGGCTTTGAGGTTACTCGTGTGCGTCCTTGTGTAGCACCGAAGTAATAc                                                                                                        >  1:79709/1‑301 (MQ=255)`  
                                                                                                                                                                                                        `cTTCACATCTTGCTCAGAGTGGTGCCGAAAGATGTGGGAAGAGACATTCGACGATGCGTACATCAAGCTGTATGAACTTTGGAAATCGAGAGGTCAATGACTATGTCAAACGTAAATACA‑GTTCACTTAGTGTGGACAATAAGAAGTTTTGGGCTACCGTAGAGTCCTCGGAGCATTCCTTCGAGGTTCCAATCTACGCTGAGACCCTAGACGAAGCTCTGGAGTTAGCCGAATGGCAATACGTTCCGGCTGGCTTTGAGGTTACTCGTGTGCGTCCTTGTGTAGCACCGAAGTAATACGa                                                                                                      >  1:179589/1‑301 (MQ=255)`  
                                                                                                                                                                                                         `ttCACATCTTGCTCAGAGGGGTGCCGAAAGATGTGGGAAGAGACATTCGACGATGCGTACATCAAGCTGTATGAACTTTGGAAATCGAGAGGTCAATGACTATGTCAAACGTAAATACAGGTTCACTTAGTGTGGACAATAAGAAGTTTTGGGCTACCGTAGAGTCCTCGGAGCATTCCTTCGAGGTTCCAATCTACGCTGAGACCCTAGACGAAGCTCTGGAGTTAGCCGAATGGCAATACGTTCCGGCTGGCTTTGAGGTTACTCGTGTGCGTCCTTGTGTAGCACCGAAGTAATACGa                                                                                                      <  1:44988/301‑1 (MQ=255)`  
                                                                                                                                                                                                          `tCACATCTTGCTCAGAGTGGTGCCGAAAGATGTGGGAAGAGACATTCGACGATGCGTACATCAAGCTGTATGAACTTTGGAAATCGAGAGGTCAATGACTATGTCAAACGTAAATACAGGTTCACTTAGTGTGGACAATAAGAAGTTTTGGGCTACCGTAGAGTCCTCGGAGCATTCCTTCGAGGTTCCAATCTACGCTGAGACCCTAGACGAAGCTCTGGAGTTAGCCGAATGGCAATACGTTCCGGCTGGCTTTGAGGTTACTCGTGTGCGTCCTTGTGTAGCACCGAAGTAATACGAc                                                                                                     <  1:43293/301‑1 (MQ=255)`  
                                                                                                                                                                                                                    `ctaagagTGGTGGCGAAAGATGTGGGAAGAGACATTAGACGATGCGTACATCAAGCTGTATGAACTTTGGAAATCGAGAGGTCAATGACTATGTCAAACGTAAATACAGGTTCACTTAGTGTGGACAATAAGAAGTTTTGGGCTACCGTAGAGTCCTCGGAGCATTCCTTCGAGGTTCCAATCTACGCTGAGACCCTAGACGAAGCTCTGGAGTTAGCCGAATGGCAATACGTTCCGGCTGGCTTTGAGGTTACTCGTGTGCGTCCTTGTGTAGCACCGAAGTAATACGACTCACTATTAg                                                                                           <  1:4801/298‑1 (MQ=255)`  
                                                                                                                                                                                                                      `gagacaggtgCCGAAAGATGTGGGAAGAGACATTCGACGATGCGTACATCAAGCTGTATGAACTTTGGAAATCGAGAGGTCAATGACTATGTCAAACGTAAATACAGGTTCACTTAGTGTGGACAATAAGAAGTTTTGGGCTACCGTAGAGTCCTCGGAGCATTCCTTCGAGGTTCCAATCTACGCTGAGACCCTAGACGAAGCTCTGGAGTTAGCCGAATGGCAATACGTTCCGGCTGGCTTTGAGGTTACTCGTGTGCGTCCTTGTGTAGCACCGAAGTAATACGACTCACTATTAggg                                                                                         <  1:309079/295‑1 (MQ=255)`  
                                                                                                                                                                                                                      `cAGAGTGGTGCCGAAAGATGTGGGAAGAGACATTCGACGATGCGTACATCAAGCTGTATGAACTTTGGAAATCGAGAGGTCAATGACTATGTCAAACGTAAATACAGGTTCACTTAGTGTGGACAATAAGAAGTTTTGGGCTACCGTAGAGTCCTCGGAGCATTCCTTCGAGGTTCCAATCTACGCTGAGACCCTAGACGAAGCTCTGGAGTTAGCCGAATGGCAATACGTTCCGGCTGGCTTTGAGGTTACTCGTGTGCGTCCTTGTGTAGCACCGAAGTAATACGACTCACTATTAggg                                                                                         <  1:343816/301‑1 (MQ=255)`  
                                                                                                                                                                                                                      `cAGAGTGGTGCCGAAAGATGTGGGAAGAGACATTCGACGATGCGTACATCAAGCTGTATGAACTTTGGAAATCGAGAGGTCAATGACTATGTCAAACGTAAATACAGGTTCACTTAGTGTGGACAATAAGAAGTTTTGGGCTACCGTAGAGTCCTCGGAGCATTCCTTCGAGGTTCCAATCTACGCTGAGACCCTAGACGAAGCTCTGGAGTTAGCCGAATGGCAATACGTTCCGGCTGGCTTTGAGGTTACTCGTGTGCGTCCTTGTGTAGCACCGAAGTAATACGACTCACTATTAggg                                                                                         <  1:224545/301‑1 (MQ=255)`  
                                                                                                                                                                                                                        `gagTGGTGCCGAAAGATGTGGGAAGAGACATTCGACGATGCGTACATCAAGCTGTATGAACTTTGGAAATCGAGAGGTCAATGACTATGTCAAACGTAAATACAGGTTCACTTAGTGTGGACAATAAGAAGTTTTGGGCTACCGTAGAGTCCTCGGAGCATTCCTTCGAGGTTCCAATCTACGCTGAGACCCTAGACGAAGCTCTGGAGTTAGCCGAATGGCAATACGTTCCGGCTGGCTTTGAGGTTACTCGTGTGCGTCCTTGTGTAGCACCGAAGTAATACGACTCACTATTAGGGaa                                                                                       <  1:50864/301‑1 (MQ=255)`  
                                                                                                                                                                                                                                 `caaagaaTGGGGGAAGAGACATTAGACGATGCGTACATCAAGCTGTATGAACTTTGGAAATCGAGAGGTCAATGACTATGTCAAACGTAAATACAGGTTCACTTAGTGTGGACAATAAGAAGTTTTGTGCTACCGTAGAGTCCTCGGAGCATTCCTTCGAGGTTCCAATCTACGCTGAGACCCTAGACGAAGCTCTGGAGTTAGCCGAATGGCAATACGTTCCGGCTGGCTTTGAGGTTACTCGTGTGCGTCCTTGTGTAGCACCGAAGTAATACGACTCACTATTAGGGAAGACTCCctc                                                                              <  1:320684/295‑1 (MQ=255)`  
                                                                                                                                                                                                                                     `aGATGTGGGAAGAGACCTTCGACGATGGTTACATCAAGCTGTATGAACTTTGGAAATCGAGAGGTCAATGACTATGTCAAACGTAAATACAGGTTCACTTAGTGTGGACAATAATAAGTTTTGGGCTACCGTAGAGTCCTCGGAGCATTCCTTCGAGGTTCCAATCTACGCTGAGACCCTAGACGAAGCTCTGGAGTTAGCCGAATGGCAATACGTTCCGGCTGGCTTTGAGGTTACTCGTGTGCGTCCTTGTGTAGCACCGAAGTAATACGACTCACTATTAGGGAAGACTCCCTCTgag                                                                          <  1:248379/301‑1 (MQ=255)`  
                                                                                                                                                                                                                                     `aGATGGAGGAAGAGACATTCGACGATGCGTAGATCAAGCTGTATGAACTTTGGAAATCGAGAGGTCAATGACTATGTCAAACGTAAATACAGGTTCACTTAGTGTGGACAATAAGAAGTTTTGGGCTACCGTAGAGTCCTCGGAGCATTCCTTCGAGGTTCCAATCTACGCTGAGACCCTAGACGAAGCTCTGGAGTTAGCCGAATGGCAATACGTTCCGGCTGGCTTTGAGGTTACTCGTGTGCGTCCTTGTGTAGCACCGAAGTAATACGACTCACTATTAGGGAAGACTCCCTCTgag                                                                          <  1:185493/301‑1 (MQ=255)`  
                                                                                                                                                                                                                                                   `aCATTCGACGATGCGTACATCAAGCTGTATGAACTTTGGAAATCGAGAGGTCAATGACTATGTCAAACGTAAATACAGGTTCACTTAGTGTGGACAATAAGAAGTTTTGGGCTACCGTAGAGTCCTCGGAGCATTCCTTCGAGGTTCCAATCTACGCTGAGACCCTAGACGAAGCTCTGGAGTTAGCCGAATGGCAATACGTTCCGGCTGGCTTTGAGGTTACTCGTGTGCGTCCTTGTGTAGCACCGAAGTAATATGACTCACTATTAGGGAAGACTCCCTCTGAGACACCAAACGAAAc                                                            >  1:323406/1‑301 (MQ=255)`  
                                                                                                                                                                                                                                                   `aCATTCGACGATGCGTACATCAAGCTGTATGAACTTTGGAAATCGAGAGGTCAATGACTATGTCAAACGTAAATACAGGTTCACTTAGTGTGGACAATAAGAAGTTTTGGGCTACCGTAGAGTCCTCGGAGCATTCCTTCGAGGTTCCAATCTACGCTGAGACCCTAGACGAAGCTCTGGAGTTAGCCGAATGGCAATACGTTCCGGCTGGCTTTGAGGTTACTCGTGTGCGTCCTTGTGTAGCACCGAAGTAATACGACTCACTATTAgggctgtctcttatacacatctccgagcccac                                                            >  1:12679/1‑272 (MQ=255)`  
                                                                                                                                                                                                                                                    `cATTCGACTATGCTTACATCAAGCTGTATGAACTTTGGAAATCGAGAGGTCAATGACTATGTCAAACGTAAATACAGGTTCACTTAGTTTGGACAATAAGAAGTTTTGGGCTACCGTAGAGTCCTCGGAGCATTCCTTCGAGGTTCCAATCTACGCTGAGACCCTAGACGAAGCTCTGGAGTTAGCCGAATGGCAATACGTTCCGGCTGGCTTTGAGGTTACTCGTGTGCGTCCTTGTGTAGCACCGAAGTAATACGACTCACTATTAGGGAAGACTCCCTCTGAGAAACCAAACGAAAcc                                                           <  1:32941/301‑1 (MQ=255)`  
                                                                                                                                                                                                                                                          `tgtataagagacagaaGCTGTATGAACTTTGGAAATCGAGAGGTCAATGACTATGTCAAACGTAAATACAGGTTCACTTAGTGTGGACAATAAGAAGTTTTGGGCTACCGTAGAGTCCTCGGAGCATTCCTTCGAGGTTCCAATCTACGCTGAGACCCTAGACGAAGCTCTGGAGTTAGCCGAATGGCAATACGTTCCGGCTGGCTTTGAGGTTACTCGTGTGAGTACTTGTGTAGCATCGAAGTAATACGACTCACTATTAGGGAAGACTCCCTCTGAGAAACCAAACGAAACCTAAAgg                                                     <  1:132209/287‑1 (MQ=255)`  
                                                                                                                                                                                                                                                                         `aGCTGTATGAACTTTGGAAATCGAGAGGTCAAGGACTATGTCAAACGTAAATACAGGTTCACTTAGTGTTGACAATAAGAAGTTTTGGGCTACCGTAGAGTCCTCGGAGCATTCCTTCGAGGTTCCAATCTACGCTGAGACCCTAGACGAAGCTCTGGAGTTAGCCGAATGGCAATACGTTCCGGCTGGCTTTGAGGTTACTCGTGTGCGTCCTTGTGTAGCACCGAAGTAATACGACTCACTATTAGGGAAGACTCCCTCTGAGAAACCAAACGAAACCTAAAGGAGATTAACATTATgg                                      <  1:259345/301‑1 (MQ=255)`  
                                                                                                                                                                                                                                                                          `gCTGTATGAACTTTGGAAATCGAGAGGTCAATGAATATGTCAAACGTAAATACAGGTTAACGTAGTGTGGACAATAAGAAGTTTTGGGCTACCGTAGAGTCCTCGGAGCATTCCTTCGAGGTTCCAATCTACGCTGAGACCCTAGACGAAGCTCTGGAGTTAGCCGAATGGCAATACGTTCCGGCTGGCTTTGAGGTTACTCGTGTGCGTCCTTGTGTAGCACCGAAGTAATACGACTCACTATTAGGGAAGACTCCCTCTGAGAAACCAAACGAAACCTAAAGGAGATTAACATTATGGc                                     <  1:10982/301‑1 (MQ=255)`  
                                                                                                                                                                                                                                                                             `gTATGAACTTTGGAAATCGAGAGGTCAATGACTATGTCAAACGTAAATACAGGTTCACTTAGTGTGGACAATAAGAAGTTTTGGGCTACCGTAGAGTCCTCGGAGCATTCCTTCGAGGTTCCAATCTACGCTGAGACCCTAGACGAAGCTCTGGAGTTAGCCGAATGGCAATACGTTCCGGCTGGCTTTGAGGTTACTCGTGTGCGTCATTGTGTAGCACCGAAGTAATACGACTCACTATTAGGGAAGACTCCCTCTGAGAAACCAAACGAAACCTAAAGGAGATTAACATTATGGCTaa                                  >  1:161341/1‑301 (MQ=255)`  
                                                                                                                                                                                                                                                                                            `aTCGAGAGGTCAATGACTATGTCAAACGTAAATACAGGTTCACTTAGTGTGGACAATAAGAAGGTTTGGGCTACCGTAGAGTCCTCGGAGCATTCCTTCGAGATTCCAATCTACGCTGAGACCCTAGACGAAGCTCTGGAGTTAGCCGAAAGGCAATACGTTCCGGCTGGCTTTGAGGTTACTCGTGTGCGTCCTTGTGTAGCACCGAAGTAATACGACTCACTATTAGGGAAGACTCCCTCTGAGAAACCAAACGAAACCTAAAGGAGATTAACATTATGGCTAAGAAGATTTTCACctc                   <  1:327020/301‑1 (MQ=255)`  
                                                                                                                                                                                                                                                                                                   `ggTCACTGACTATGTCAAACGTAAATACAGGTTCACTTAGTGTGGACAATAAGAAGTTTTTGGCTACCGTAGAGTCCTCGGAGCATTCCTTCGAGGTTCCAATCTACGCTGAGACCCTAGACGAAGCCCTGGAGTTAGCCGAATGGCAATACGTTCCGGCTGGCTTTGAGGTTACTCGTGTGCGTCCTTGTGTAGCACCGAAGTAATACGACTCACTATTAGGGATGACTCCCTCTGAGAAACCAAACGAAACCTAAAGGAGATTTACATTATGGCTAAGACGATTTTCACCTCTGCGCTg            >  1:75499/1‑301 (MQ=255)`  
                                                                                                                                                                                                                                                                                                      `cAATGACTATGTCAAACGTAAATACAGGTTCACTTAGTGTGGACAATAAGAAGTTTTGGGCTACCGTAGAGTCCTCGGAGCATTCCTTCGAGGTTCCAATCTACGCTGAGACCCTAGACGAAGCTCTGGAGTTAGCCGAATGGCAATACGTTCCGGCTGGCTTTGAGGTTACTCGTGTGCGTCCTTGTGTAGCACCGAAGTAATACGACTCACTATTAGGGAAGACTCCCTCTGAGAAACCAAACGAAACCTAAAGGAGATTAACATTATGGCTCAGAAGATTTTCACCTCTGCGCTGGGt         >  1:100077/1‑301 (MQ=255)`  
                                                                                                                                                                                                                                                                                                        `cagACTATTTCAAACGTAAATACAGGTTCACTTAGTGTGGACAATAAGAATTTTTGGTCTACCTTAGAGTCCTCGGAGCATTCCTTCGAGTTTCCAATCTACGCTGAGACCCTAGACGAAGCTCTGGAGTTAGCCGAATGGCAATACGTTCCGTCTGGCTTTGAGGTTACTCGTGTGCGTCCTTGTGTAGCACCGAAGTAATACGACTCACTATTAGGGAATACTCCCTCTGAGAAACCAAACGAAACCTAAAGGAGATTAACATTATTGCTAAGAAGATTTTCACCTCTGCGCTGGGTAc       <  1:56625/299‑1 (MQ=255)`  
                                                                                                                                                                                                                                                                                                         `tGACTATGTCAAACGTAAATACAGGTTCACTTAGTGTGGACAATAAGAAGTTTTGGGCTACCGTAGAGTCCTCGGAGCATTCCTTCGAGGTTCCAATCTACGCTGAGACCCTAGACGAAGCTCTGGAGTTAGCCGAATGGCAATACGTTCCGGCTGGCTTTGAGGTTACTCGTGTGCGTCCTTGTGTAGCACCGAAGTAATACGACTCACTATTAGGGAAGACTCCCTCTGAGAAACCAAACGAAACCTAAAGGAGATTAACATTATGGCTAAGAAGATTTTCACCTCTGCGCTGGGTAcc      >  1:12989/1‑301 (MQ=255)`  
                                                                                                                                                                                                                                                                                                           `aCTATGTCAAACGTAAATACAGGTTCACTTAGTGTGGACAATAAGAAGTTTTGGGCTACCGTAGAGTCCTCGGAGCATTCCTTCGAGGTTCCAATCTACGCTGAGACCCTAGACGAAGCTCTGGAGTTAGCCGAATGGCAATACGTTCCGGCTGGCTTTGAGGTTACTCGTGTGCGTCCTTGTGTAGCACCGAAGTAATACGACTCACTATTAGGGAAGACTCCCTCTGAGAAACCAAACGAAACCTAACGGAGATTAACATTATGGCTAAGAAGATTTTCACCTCTGCGCTGGGTACCGc    <  1:137076/301‑1 (MQ=255)`  
                                                                                                                                                                                                                                                                                                           `aCTATGTCAAACGTAAATACAGGGTCACTTAGTGTGGACAATAAGAAGTTTTGGGCTACCGTAGAGTCCTCGGAGCATTCCTTCGAGGTTCCAATCTACGCTGAGACCCTAGACGAAGCTCTGGAGTTAGCCGAATGGCAATACGTTCCGGCTGGCTTTGAGGTTACTCGTGTGCGTCCTTGTGTAGCACCGAAGTAATACGACTCACTATTAGGGAAGACTCCCTCTGAGAAACCAAACGAAACCTAAAGGAGATTAACATTATGGCTAAGAAGATTTTCACCTCTGCGCTGGGTACCGc    <  1:87168/301‑1 (MQ=255)`  

                                                                                                                                                                                                                                                                                                             `|`                                                                                                                                                                                                                                                                                                              
`GACCGTTGAGCAGTTCAAGGGATACTGCTTCGGTAACATCTTAAAGTACAGACTACGTGCTGGTAAGAAGTCAGAGTTAGCGTACTTAGAGAAAGACCTAGCGAAAGCAGACTTCTATAAAGAACTCTTTGAGAAACATAAGGATAAATGTTATGCATAACTTCAAGTCAA‑CCCCACCTGCCGACAGCCTATCTGATGACTTCACATCTTGCTCAGAGTGGTGCCGAAAGATGTGGGAAGAGACATTCGACGATGCGTACATCAAGCTGTATGAACTTTGGAAATCGAGAGGTCAATGACTATGTCAAACGTAAATACAGGTTCACTTAGTGTGGACAATAAGAAGTTTTGGGCTACCGTAGAGTCCTCGGAGCATTCCTTCGAGGTTCCAATCTACGCTGAGACCCTAGACGAAGCTCTGGAGTTAGCCGAATGGCAATACGTTCCGGCTGGCTTTGAGGTTACTCGTGTGCGTCCTTGTGTAGCACCGAAGTAATACGACTCACTATTAGGGAAGACTCCCTCTGAGAAACCAAACGAAACCTAAAGGAGATTAACATTATGGCTAAGAAGATTTTCACCTCTGCGCTGGGTACCGCTG  >  NC_001604.1/8597‑9197`

  

Alignment Legend

Aligned base mismatch/match (shaded by quality score): `ATCG/ATCG` `< 3 ≤` `ATCG/ATCG` `< 13 ≤` `ATCG/ATCG` `< 27 ≤` `ATCG/ATCG` `< 36 ≤` `ATCG/ATCG` `< 38 ≤` `ATCG/ATCG`

Unaligned base: `atcg`    Masked matching base: `atcg`    Alignment gap: `‑`    Deleted base: `‑`

Reads not counted as support for junction

read\_name Not counted due to insufficient overlap past the breakpoint.

read\_name Not counted due to not crossing MOB target site duplication.
